# Diagnosis and Prognostic Analysis of *Mycoplasma pneumoniae* Pneumonia in Children Based on High-Resolution Computed Tomography

**DOI:** 10.1155/2022/1985531

**Published:** 2022-04-22

**Authors:** Jiangang Leng, Zemin Yang, Wenhui Wang

**Affiliations:** Department of Pediatrics, Funan County People's Hospital, Funan County, Fuyang City 236300, Anhui Province, China

## Abstract

Mycoplasma *pneumoniae* (MP) is defined as a common cause of pulmonary infections and accounts for up to four over ten of pneumonia in children over age 5. This study was aimed to explore the diagnosis and prognosis of mycoplasma *pneumoniae* pneumonia (MPP) in children using high-resolution computed tomography (CT) (HRCT). 71 children hospitalized with MPP were undertaken as the research objects to observe the incidence rate, occurrence time, and duration of the clinical symptoms and pathological signs. The chest HRCT and pulmonary ventilation function (PVF) were examined in the acute phase, the second phase re-examination period, and the third phase re-examination period. Relevant indicators were statistically analyzed to determine the change rules of chest HRCT and PVF and correlation between the two. Clinically, the children with MPP suffered from fever, cough, and sore throat. In addition to the above symptoms, children with MPP had different degrees of PVF impairment. Compared with the group with normal HRCT results, the forced vital capacity (FVC), forced expiratory volume in 1 second (FEV1), peak expiratory flow (PEF), forced expiratory flow at 25% forced expiratory volume (FEF25), forced expiratory flow at 50% forced expiratory volume (FEF50), forced expiratory flow at 75% forced expiratory volume (FEF75), and maximum mid-expiratory flow (MMEF75/25) of children in bronchopneumonia group, segmental pneumonia group, and lobar pneumonia group were obviously reduced, showing statistically great differences (*P* < 0.05). Compared with the case in acute phase, the PVF indicators of children in the re-examination phases were much higher, with greatly statistical differences (*P* < 0.05). In children with MPP, both the large and small airways were affected, but the recovery of the small airways was slow. Pulmonary HRCT and PVF can be undertaken as important indicators to judge the severity and prognosis of MPP in school-age children.

## 1. Introduction

Mycoplasma *pneumoniae* (MP) is well-known as an intracellular pathogen that can cause respiratory diseases and extra-pulmonary diseases of children, which is commonly named as mycoplasma *pneumoniae* pneumonia (MPP). The diseases are mostly spread by respiratory droplets, and sporadic infections occur throughout the year, especially in late autumn and early winter [1]. The incidence rate of MP infection has shown an upward trend year by year, which has been proved by related studies in recent years. Date for MPP in China is scarce. Data show that MP infection accounts for 10–40% of community-acquired pneumonia (CAP) in children at 9–14 years old, with a peak age of 4–6 years [2]. The clinical manifestations and chest X-ray examination of MPP are not characteristic, and the diagnosis cannot be made based on the clinical manifestations and chest X-ray examination alone. For a definitive diagnosis, testing for the pathogen is required. At present, the diagnosis of MPP in China mainly relies on serological tests. However, reports vary by age, region, year, and year of prevalence, so there are various reports with similar results. MPP has been well recognized as one of the most critical and dangerous health problems all over the China. With the changes in etiology, MPP shows new epidemiological characteristics, manifested as specific imaging changes that affect the pulmonary ventilation function (PVF), but the obvious pathogenesis is still unclear [3]. Current research believes that the pathogenesis of MPP is related to cell damage and immune inflammatory response caused by direct invasion, but the degree of damage and the duration of action are still unclear, and the clinical manifestations are often atypical. Studies have shown that the major and minor respiratory tract functions of children with MPP in acute phase have varying degrees of damage, the functions of both large and small airways are damaged in different degrees in children, especially when the MPP is in the acute phase. In addition, the lung function of most children can be improved significantly during the recovery period, but the PVF in small airway is more severe and slower to be recovered. Patients with markedly higher MP antibody titers may have more severe PVF impairment. The PVF impairments could be improved greatly after treatment, so they are reversible. MPP can induce asthma attacks in some patients with asthma. In addition, acute MP infection can not only promote acute attacks in children with asthma but also cause wheezing in nonasthmatic children [4]. MPP is mainly treated with antibiotics. Since cough is the most prominent clinical manifestation, low-dose antitussives and expectorants can be given appropriately. Those with severe symptoms of hypoxia should be given oxygen in time. For severe asthma, bronchodilators can be used. Adrenal corticosteroids can be used for patients with rapid and severe mycoplasma pneumonia in the acute stage or persistent pulmonary lesions resulting in atelectasis, pulmonary fibrosis, bronchiectasis, or extra-pulmonary complications.

Many new information on MPP infections is published in the last several years. Many studies have linked MP infection to asthma attacks. Therefore, MPP is currently considered to be an important cause of asthma exacerbations in nonasthmatic children [5]. Studies have shown that the abnormal rate of lung imaging of MPP patient is observably higher in contrast to the positive rate of lung signs. The lung imaging of patients with severe MPP often presents as large patchy shadows, possible atelectasis, pleural effusion, necrotizing pneumonia, pulmonary abscesses, and other pulmonary complications [6]. School-age children account for a higher proportion of MPP patients, with severe and diverse clinical manifestations and different degrees of PVF impairment [7]. Acute MPP can be developed into bronchiolitis obliterans if it is not controlled well in children, which can greatly affect the quality of life of children and is associated with MPP. Early identification and treatment of MPP is conductive to preventing the development of bronchiolitis obliterans. MP infection is impossible to be identified or confirmed based on a single clinical characteristic only. However, if the duration of pyrexia and severe fever is long, the blood oxygen saturation is reduced, and the ALT and LDH are increased, it can be determined as the MP infection. On the other hand, MP infection can be diagnosed by high-resolution computed tomography (HRCT) examination, because it can observe lung lesions from multiple levels, and it is easy to accurately locate and clarify the degree and scope of lesions. Pulmonary function suggests severe MPP involving the large airways, with concomitant stenosis and occlusion of the lower small airways [8]. Lung structural abnormalities are common in school-aged children after MPP surgery, but there are few studies on imaging, especially HRCT, and their correlation with PVF during the course of the disease (CoD). HRCT and PVF follow-up should be performed on school-age children with MPP at a fixed time. Chest HRCT and PVF test can be undertaken as important reference indicators for the diagnosis and prognosis of MPP [9]. Exploring the change rules of chest HRCT and PVF in children with MPP and correlation between the two can provide important support for clinical diagnosis, condition evaluation, treatment guidance, and prognosis judgment.

## 2. Materials and Methods

### 2.1. Objects

Seventy-one children were selected who admitted to hospital from October 2019 to December 2021. Inclusion criteria were given as follows: the patients were preschool and school-age children; children with MPP in acute onset, with a duration of less than 5 days on admission, accompanied by fever and respiratory symptoms; children whose X-ray examination of the chest showed patchy or patchy infiltrates or interstitial changes, with or without pleural effusion; and children with positive (+) result of serum MP-IgM antibody (diluted 1 : 39) diluted using microparticle agglutination method. Exclusion criteria were set as follows: children with autoimmune diseases and other chronic diseases and were taking some drugs that affected immune function; patients with respiratory diseases such as *tuberculosis* and previous bronchial asthma; patients with mixed infections determined by virus testing, sputum culture, and blood culture, and so on; and children whose parents refused to accept this experiment. Discontinuation criteria were set as follows: serious adverse events and feelings occurred during the treatment period; and the loss rate of subjects during the experiment was >19%. The experiment here complied with the ethical requirement and all children and their families had been aware of experimental procedure and signed the agreements.

### 2.2. CT Scanning

HRCT is believed to observe the fine structure of lesions, and it is an ideal supplement to conventional chest scans. HRCT examination was performed with 258-slice microplate rapid CT. The scanning voltage was 122 kV, and the fault was 1–1.8 mm. After diagnosis and treatment, the lung HRCT examination was performed again. It should scan continuously from the lung tip to the diaphragm. Before the HRCT examination, the patients were instructed with some key points to cooperate with the examinations. After the HRCT examination, the distribution and morphological characteristics of lung, pleura, and mediastinum were evaluated carefully by radiologists.

### 2.3. Treatment Methods and Follow-Up

The treatment included macrolide antibiotics (referring to pediatric medication methods); symptomatic treatment (like aerosol inhalation); bronchoscopy for lobar or segmental pneumonia according to the situation; and adrenal cortex hormone or gamma globulin for MPP at the extreme stage of inflammation. Patients who met the following conditions were discharged after oral medication and sequential treatment. If the cough was obviously relieved, the general condition was better; if the auxiliary temperature was <37.6°C for 72 h; and chest X-rays showed improvement in absorption. Patients with severe PVF impairment caused by MPP had to be supplied with oxygen in time. For patients with mild PVF impairment, bronchodilators can be used. Adrenal corticosteroids can be used for patients with rapid and severe MPP in the acute stage or persistent pulmonary lesions resulting in atelectasis, pulmonary fibrosis, bronchiectasis, or extra-pulmonary complications. The total course of treatment was 14–28 days and the follow-up was 3–6 months.

### 2.4. PVF Test (FVC Method)

The environmental parameters and volume calibrations of the lung function instrument were checked before the examination. The predicted value was automatically generated by the PVF instrument software according to age, height, weight, and other basic parameters. The patient should keep his/her head stood naturally horizontally with the mouth closed, inhale deeply to the total lung volume (TLC) level, and maintain the exhalation to the functional residual capacity (FRC) level with the maximum volume and the fastest speed. In addition, it should observe the time-volume curve and flow-volume curve. The best test value should be kept three times (each time the difference was required to be <5% or < 0.2 L) to get the average value as the judgment result. The abovementioned operations were implemented by specialized staff trained in pulmonary function measurement so that the operations were professional enough and could be implemented correctly. In addition, the patient was trained to cooperate with the test in advance so that the test could be completed as smooth as possible.

### 2.5. Observation Indicators

The observation indicators included forced vital capacity (FVC), forced expiratory volume in 1 second (FEV1), peak expiratory flow rate (PEF), forced expiratory flow at 25% forced expiratory volume (FEF25), forced expiratory flow at 50% forced expiratory volume (FEF50), forced expiratory flow at 75% forced expiratory volume (FEF75), and maximum mid-expiratory flow (MMEF75/25). The PVF test results were as follows: first, the percentage of the FEV1 to the predicted value (FEV1%) < 79% indicated obstructive ventilation dysfunction (OVDF); FVC % < 79% suggested restrictive ventilation dysfunction (RVDF), and FEV1% < 79% and FVC% < 79% indicated mixed ventilation dysfunction (MVDF). Second, MMEF, FEF50%, FEF75%, and MMEF 75/25 of <80% indicated that the ventilation function of the small airway was affected. If FEV1, PEF, and FEF25 were normal, while FEF50 and FEF75 decreased <79%, it indicated early OVDF of small airway.

### 2.6. Statistical Analysis

Spss18 was adopted to complete statistics. The test results were expressed as mean ± standard deviation. The independent sample *t*-test and one-way analysis of variance were adopted between groups, and the test level was *a* = 0.05. *P* < 0.05 suggested the difference was obvious statistically.

## 3. Results

### 3.1. Basic Information of the Children

**Figure 1:**
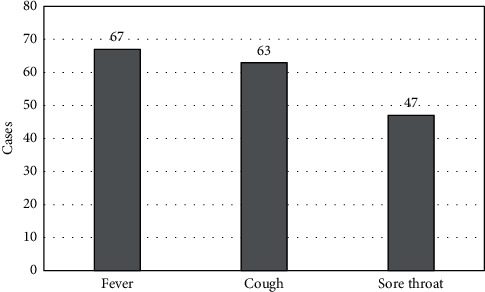
Statistics of the main clinical manifestations of children.

The clinical manifestations of 71 school-age children with MPP were analyzed. Among them, there were 37 males and 34 females; the mean age, height, and weight were 8.55 ± 1.84 years old, 141 ± 7.8 cm, and 28.9 ± 6.4 kg, respectively. PVF test was performed in 71 cases in acute stage (with CoD of 7.5 ± 2.1 days) and 62 cases in re-examination stages (with CoD of 30.2 ± 3.8 days). The HRCT was performed on 57 cases in acute stage (CoD was 7.5 ± 2.0 days) and 52 cases in re-examination stages (CoD was 30.2 ± 4.3 days). The main symptoms were fever (67/94.4%), cough (63/88.7%), and sore throat (47/66.2%). The specific data were shown in Figure 1. Among the clinical manifestations, fever appeared earliest and most frequently, with an average CoD of 1.48 ± 0.83 days; while cough lasted the longest (the average CoD was 11.51 ± 1.55 days). In addition, early lung signs were absent, and some cases may have transient wheezing and wet rales in the lungs, and may be accompanied by extra-pulmonary symptoms.

### 3.2. CT Image Characteristics of Children before and after Treatment

**Figure 2:**
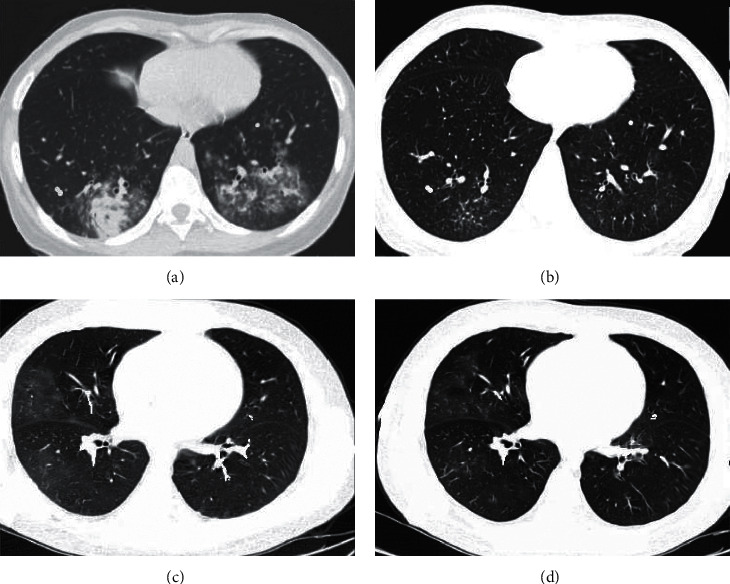
CT image characteristics of children before and after treatment.

The chest CT images of the children before and after treatment were analyzed and compared, and the results were shown in Figure 2. The 1# child had high fever and severe cough before treatment, the MP-IgM antibody was 3.6 (positive >1.11), and only Azithromycin and other drugs (which were the commonly adopted drugs for MPP) were taken for clinical antibacterial treatment. The above clinical symptoms were found to decrease greatly after treatment with above operations. It indicated that Azithromycin was very effective to 1# child with MPP. Figures 2(a) and 2(b) showed that the CT images before and treatment, respectively. The 2# child claimed high fever, cough, and chest pain before treatment. After treatment with Moxifloxacin and other drugs, it was found that the absorption of the lesion improved to varying degrees. It meant that Moxifloxacin was effective for 2# child.

**Figure 3:**
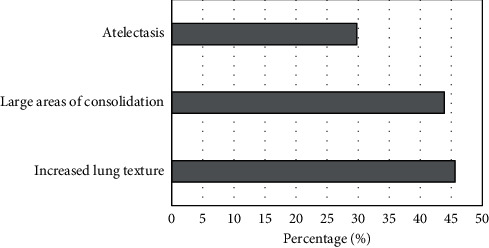
The main manifestations of chest HRCT in children with MPP in acute phase.

The chest HRCT manifestations of school-age children with MPP showed diversity, including increased lung texture, rod-shaped shadow, ground glass shadow, small patchy shadow, large patchy consolidation, atelectasis, bronchiectasis, chest cavity effusion, and hilar mediastinal lymph nodes swelling. In acute phase group, multiple lung lobes were involved more than a single lung lobe; while in the re-examination groups, more single lung lobes were found than multiple lung lobes, and the lesions in each group mainly involved the right lower lung. Increased lung texture was the most common in acute phase (26 cases, 45.6%). In addition, there were 25 cases with large-scale consolidation (accounting for 43.9%), and there were 17 children with atelectasis (29.8%), as shown in Figure 3. The lesions in the re-examination group had various degrees of absorption, of which 36 children (69.2%) had full absorption of the lesions. Figure 3 illustrated the specific results with exact data.

### 3.3. Comparison of PVF Indicators in Children with Different HRCT Manifestations

**Figure 4:**
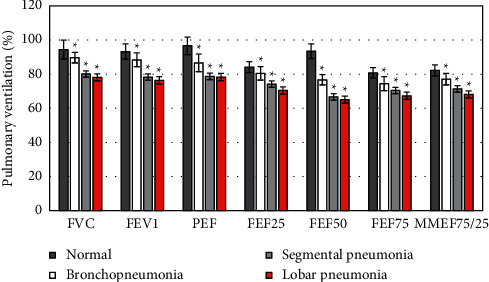
The correlation between different lung HRCT manifestations and PVF in children ( ^*∗*^(P) < 0.05).

Based on the HRCT manifestations of MPP children, they were rolled into three different groups: a bronchopneumonia group, a segmental pneumonia group, and a lobar pneumonia group. The HRCT manifestations were different, which meant that the severity of the MPP was not the same, so children in each group had different degrees of PVF impairment. The OVDF was the major symptom in the bronchopneumonia group, which was found in large airways of the affected children. In the segmental pneumonia and lobar pneumonia groups, children were manifested as MVDF, which was concentrated on their small airways. The differences in PVF indicators of children with different manifestations were compared (as illustrated in Figure 4). It can be known that compared with the group with normal HRCT manifestations, all the PVF indicators in bronchopneumonia group, segmental pneumonia group, and lobar pneumonia group were all reduced, with statistically remarkable differences (*P* < 0.05). In addition, all above PVF indicators in children in lobar pneumonia group were always the lowest in contrast to the segmental pneumonia group and the bronchopneumonia group.

### 3.4. Comparison of PVF Indicators of Children before and after Treatment

**Figure 5:**
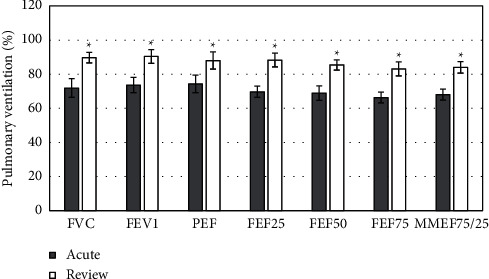
Comparison of PVF indicators in MMP children at different stages ( ^*∗*^(P) < 0.05).

School-age children with MPP showed different degrees of PVF impairment, of which OVDF was dominated. The changes of PVF indicators in different phases were compared, and the results were analyzed and displayed in Figure 5. From results in Figure 5, it can be observed that compared to the acute phase, the PVF indicators of children in the re-examination period increased sharply, and statistically visible differences could be found (*P* < 0.05).

## 4. Discussion

MPP is an acute lung inflammation caused by MP infection and is a disease commonly found in children's respiratory system. It is often accompanied by atelectasis and massive lung infiltration, which can cause extra-pulmonary complications. MPP can cause children to suffer from some flu-like symptoms, which can increase the burden on their health. The incidence of MP infection in children has shown an upward trend these years, and data show that MP infection accounts for 11%–41% of CAP in children, which may be even higher [10]. MP infection is a pathogen of pediatric respiratory infections in infants and adolescents, most of which are clinically manifested as respiratory tract infection syndrome, of which about 3% to 10% can develop into MPP. MPP is sporadic and prevalent throughout the year, with a high incidence in preschool and school-age. MPP due to MP infection is usually mild, but some cases develop a severe condition, so that the life is threatened. MPP is a frequent but underdiagnosed disease in children, and appropriate treatment cannot be given early. Patients with severe symptoms of PVF have to be intervened with oxygen supply in time. For severe PVF, bronchodilators can be used. Adrenal corticosteroids can be used for patients with rapid and severe MPP in the acute stage or persistent pulmonary lesions resulting in atelectasis, pulmonary fibrosis, bronchiectasis, or extra-pulmonary complications. MPP imaging findings in children are mostly unilateral lesions, accounting for more than 80%, mainly in the lower lobes, sometimes only in the shadow of the hilum, the shadow weight increases, irregular cloud-like lung infiltration, and the lung field extends from the hilum, especially in the lower lobes of both lungs. Both lungs may show diffuse network or nodular infiltration or interstitial pneumonia, and the lung segments or lobes are not consolidated. However, MPP has become a new epidemiological feature, due to changes in etiology, specific imaging changes affect lung function [11]. The chest radiograph has high spatial resolution and can display the overall status and the infection condition of the lung clearly. However, it is difficult to find the early or ultra-early pulmonary lesions due to the limitation of overlapping relationship. HRCT shows higher performance in displaying the fine structures of lung tissue (pulmonary lobular airways, blood vessels and interlobular septa, pulmonary interstitium, and millimeter-scale intrapulmonary nodules, etc.). Sometimes, it can display the morphological changes, which are similar to the gross specimen. Therefore, HRCT shows very high value in chest imaging examination. In addition, its another advantage is that no additional contrast enhancement is needed. HRCT uses thin-slice scanning to reduce the overlap caused by the volume effect and accurately collect lesion information [12]. Therefore, the local anatomy can be clearly displayed and the morphological changes of the small airway that cannot be detected by the lung function test can be observed. Early detection of injury, progress of injury, and improvement of injury after treatment can all be well evaluated. In addition, HRCT can be considered when the correlation between lung structure and lung function is assessed. Therefore, HRCT is a noninvasive, accurate, and reproducible imaging method [13], so it is especially beneficial for children, especially infants. The Philips 256-slice Micropanel CT (Brilliance NanoPanel iCT) can reduce the radiation dose of patients during the scanning process, which can be reduced by more than 80% compared with ordinary CT examinations. It shows obvious effect especially for infants and other radiation-sensitive populations. However, the potential risks of testing should be carefully evaluated.

In this work, the chest HRCT manifestations of school-age children with MPP showed diversity, including increased lung texture, rod-shaped shadow, ground glass shadow, small patchy shadow, large patchy consolidation, atelectasis, bronchus dilation, pleural effusion, and hilar mediastinal lymph nodes enlargement. More lung lobes were involved than a single lung lobe for children in the acute phase, while it was the opposite case for children in the re-examination periods. The lesions in each group mainly involved the right lower lung. Increased lung texture is the most common in acute phase, followed by large areas of consolidation and atelectasis. The multiple lesions have various degrees of absorption. A study on 207 children with MPP found that the most common manifestations of MPP on chest radiographs were lung parenchyma shadows (57%), large infiltrates (47.3%), and atelectasis (17.9%). A total of 75.1% of the children had different degrees of abnormal PVF, and most of them showed changes in small airway function. Such results are similar with the findings in this work. MPP chest radiographs are not specific, but they can show diffuse or reticular infiltration or consolidation. A total of 20% of patients have bilateral infiltration. Studies have observed the large and small airway ventilation indicators of MPP patients and found that they are significantly lower than expected, suggesting that respiratory mucosal injury and airflow limitation in children with MPP are caused by direct damage to the respiratory mucosa and immune damage caused by MP.

## 5. Conclusion

The clinical symptoms of MPP in school-age children were mainly fever, cough, and sore throat. In addition, it lacked positive signs in the early stage. The HRCT manifestations of MPP of school-age children were different, but there were some rules. There were varying degrees of lung ventilation function obstacles in MPP of school-age children, which was dominated by OVDF in small airways. Ventilation dysfunction in school-age children with MPP was related to the severity of HRCT manifestations. In children with MPP, both large and small airways were affected, but small airway mainly involved segmental pneumonia and lobular pneumonia, and its recovery was slowly. Pulmonary HRCT and PVF can be used as important indicators to judge the severity and prognosis of MPP in school-age children. However, sample included was less, so more children can be invited to participate in the experiment. Clinical trials should not be conducted in a single or small area, and it will get better performance by conducting it in multi-center hospitals.

## Data Availability

The data used to support the findings of this study are available from the corresponding author upon request.
